# High-Capacity Conductive Nanocellulose Paper Sheets for Electrochemically Controlled Extraction of DNA Oligomers

**DOI:** 10.1371/journal.pone.0029243

**Published:** 2011-12-15

**Authors:** Aamir Razaq, Gustav Nyström, Maria Strømme, Albert Mihranyan, Leif Nyholm

**Affiliations:** 1 The Ångström Laboratory, Department of Engineering Sciences, Nanotechnology and Functional Materials, Uppsala, Sweden; 2 The Ångström Laboratory, Department of Materials Chemistry Uppsala, Sweden; US Naval Reseach Laboratory, United States of America

## Abstract

Highly porous polypyrrole (PPy)-nanocellulose paper sheets have been evaluated as inexpensive and disposable electrochemically controlled three-dimensional solid phase extraction materials. The composites, which had a total anion exchange capacity of about 1.1 mol kg^−1^, were used for extraction and subsequent release of negatively charged fluorophore tagged DNA oligomers via galvanostatic oxidation and reduction of a 30–50 nm conformal PPy layer on the cellulose substrate. The ion exchange capacity, which was, at least, two orders of magnitude higher than those previously reached in electrochemically controlled extraction, originated from the high surface area (i.e. 80 m^2^ g^−1^) of the porous composites and the thin PPy layer which ensured excellent access to the ion exchange material. This enabled the extractions to be carried out faster and with better control of the PPy charge than with previously employed approaches. Experiments in equimolar mixtures of (dT)_6_, (dT)_20_, and (dT)_40_ DNA oligomers showed that all oligomers could be extracted, and that the smallest oligomer was preferentially released with an efficiency of up to 40% during the reduction of the PPy layer. These results indicate that the present material is very promising for the development of inexpensive and efficient electrochemically controlled ion-exchange membranes for batch-wise extraction of biomolecules.

## Introduction

The application of electronically conductive polymers, e.g. polyaniline, polythiophene and polypyrrole, in biosciences has been developing rapidly during more than two decades, particularly in the fields of controlled drug delivery, biomedical engineering and diagnostics [1]–[4]. One reason for the interest in these polymers stems from the fact that they can extract and release ions upon their oxidation and reduction. It is well-known [5] that the charge compensation upon the oxidation of conducting polymers can be taken care of either by anions entering the polymer or cations leaving the polymer, or a combined movement of both anions and cations, depending on the charge and size of the ions. In electrochemically controlled solid-phase micro extraction [6]–[12], this effect is used to perform batch-wise extraction and release of charged species by applying an electrical potential/current to a conducting solid phase extraction material in contact with the solution containing the charged species. In another approach, conducting polymer coated particles have been used as an electrochemically controlled stationary phase in a chromatographic separation system [13]–[19]. The latter technique, (i.e. electrochemically modulated liquid chromatography EMLC), has, however, not yet found widespread use most likely due to the relatively complex experimental set-up and problems associated with the packing of efficient columns (the stationary phase should be composed of uniform (2–10 µm) conductive particles with a sufficiently large (e.g. 150–200 m^2^ g^−1^) surface area.

Compared to conventional solid phase micro extraction (SPME) [20], in which a material with a fixed number of exchange sites is employed, electrochemically controlled SPME offers higher flexibility since the properties of the material and thus the number of exchange sites can be externally controlled by electrochemically controlling the charge of the material. The applicability of electrochemically controlled SPME has, however, so far been limited by the relative low capacities [7] of the available extraction materials. For conducting polymer films this problem generally stems from mass transport limitations appearing when attempting to increase the capacity of the films by increasing the film thickness [7], [21], [22]. It has thus been reported [7] that only the outermost layer of the polymer film on a planar electrode surface was active in the extraction of ions when using electrodes coated with micrometer thick films of conducting polymers. It can consequently be anticipated [21] that the ion exchange capacities of conducting polymer coatings could be increased significantly if a larger fraction of the polymer layer could be utilized. As has been reported recently [21], [23], [24], this can be achieved with materials obtained by coating thin layers of conducting polymers on high surface area cellulose substrates. Such materials should therefore be highly interesting for electrochemically controlled solid phase extractions of e.g. charged biomolecules.

Interactions between conducting polymers and biomolecules, such as dopamine [25], cochlear neurotrophines [26], the antipsychotic drug risperidone [27], adenosine triposphate (ATP) [28] and, in particular, DNA [4], [29]–[36] have been studied by many groups. In the DNA case, strands have been immobilized within the structure of the conductive polymer, e.g. polypyrrole (PPy), or on the surface of the polymer either employing adsorption [33], [37]–[39] or polymerization (chemically or electrochemically) in the presence of the DNA [29], [40]–[42]. For in situ polymerization, conductive polymer monomers substituted with DNA have also been studied [43]. The spontaneous adsorption of DNA on conducting polymers such as PPy has been investigated by several groups [30], [31], [33], [35], [37]–[39] and it has been shown [30], [32], [33], [35], [37]–[39] that the adsorption of DNA on partially oxidized conducting polymers involves a diffusion controlled replacement of the counter anions on the polymer with DNA [30], [32], [37], [38], [44]. It was also demonstrated that the amount of DNA adsorbed depends on the positive charge on the polymer (i.e. the oxidation state of the polymer) and that, at least, some of the adsorbed DNA can be competitively released upon the addition of other anions. The release rates were, however, found to be two orders of magnitude lower than the adsorption rates [37]. The latter finding is in good agreement with results [40] showing that PPy films act as cation exchangers when DNA molecules are immobilized within PPy, since the detachment of the DNA during the reduction of oxidized PPy films is slow enough to induce charge compensation by cation movement.

Electrochemically controlled extraction and release of DNA has, to the best of our knowledge, not been studied previously. It is therefore not known if the extractions can be made faster and more efficiently when the oxidation state of a conducting polymer (e.g. PPy) is controlled electrochemically compared to when chemically prepared (and only partially oxidized) films are employed. It is also not known if the extraction/release rates and yields can be increased by using a highly porous solid phase extraction material coated with a thin conformal layer of the conducting polymer, as can be anticipated based on the mass transport limitations previously found for µm thick polymer films [7]. Although not shown so far, the efficiency of the DNA release step most likely depends on the thickness of the PPy film as it is well-known that sufficiently thick PPy films doped with large anions generally serve as cation exchangers [45], [46].

In the present paper, we describe electrochemically controlled extraction and release of DNA oligomers from aqueous solutions, containing an excess of other ions, using a high surface area composite material. The material, which is composed of PPy and cellulose, was used in the form of conducting paper sheets with exceptionally high ion exchange capacities, low internal resistances, rapid ion exchange properties, and good stabilities [21], [23], [24], [47]. It is shown that DNA oligomers of varying length (i.e. containing 6, 20 and 40 bases) can be extracted and that the extracted amount is proportional to the oxidation charge used in the extraction step. The possibility of releasing the previously extracted DNA by reducing the polymer is also discussed based on the conductivity of the composite and the length of the DNA chains. The present composite material is shown to be a very promising candidate for rapid batch-wise electrochemically controlled extractions of DNA in which the composite is immersed directly in the DNA containing solution.

## Materials and Methods

### 2.1 Materials

#### 2.1.1. Chemicals

The cellulose used in this study was extracted from Cladophora algae, as previously described [48]. Pyrrole, iron (III) chloride, hydrochloric acid, sodium chloride, sodium tetraborate decahydrate (99.5%) and boric acid (99.5%) were purchased from Sigma Aldrich, Germany, and were used without further purification. The carbon fibers [C005715/1, Grade XAS, number of filaments 6000, Filament diameter 0.007 mm] were obtained from Goodfellow, UK. Buffer tablets (pH = 6.8), containing disodium hydrogen orthophosphate dehydrate and potassium dihydrogen phosphate, were purchased from Merck, Germany. The fluorophore-tagged oligonucleotides (oligomers) of varying length (i.e. containing 6, 20 and 40 bases) were synthesized by Biomers.net, Germany. The oligomers were single-stranded sequences of thymine (T) with no internal modifications and the (dT)_6_, (dT)_20_ and (dT)_40_ oligomers were tagged at the 3′-position with 6-FAM, TexRed and Cy3 fluorophores, respectively.

#### 2.1.2. PPy-cellulose composite

The PPy-cellulose composite was prepared by oxidative chemical polymerization of pyrrole monomers on cellulose nanofibers (20–30 nm thick) in the presence of iron (III) chloride at room temperature, as previously described [21]. The composite was featured with morphological and electrical properties identical to those reported previously [23], [24], [47]. The composite thus had the appearance of a black flexible paper sheet which could be cut into any shape by a pair of scissors. The composite possessed an internal specific surface area of 80 m^2^/g (as determined by BET analysis of nitrogen adsorption isotherms), an electrical conductivity of ∼1 S/cm, and a thickness of the PPy coating on the individual cellulose fibers of ∼50 nm as characterized by transmission electron microscopy (TEM) [23], [24], [47].

Prior to the extractions of DNA oligomers from solutions with varying DNA concentrations, the composite was rinsed with 0.5 M HCl to maintain a high doping level.

#### 2.1.3. Preparation of buffer solutions

The standard phosphate buffer solution (PBS; 6.3 mM, pH = 6.8) was prepared according to the manufacturer's instructions (Merck) by dissolving a tablet containing disodium hydrogen orthophosphate dehydrate (0.47 g) and potassium dihydrogen phosphate (0.47 g) in 1 L of deionized water. The borax buffer (pH = 8) was obtained by mixing 0.77 g of boric acid and 4.76 g of sodium tetraborate decahydrate in 250 mL of deionized water. The pH of the buffer was then adjusted to pH 8 by drop-wise addition of 0.1 M HCl.

### 2.2. Methods

#### 2.2.1. Electrochemically controlled extraction of DNA oligomers

The electrochemically controlled extraction and release measurements were performed in a standard three-electrode electrochemical cell utilizing an Autolab/GPES instrument (ECO Chemie, The Netherlands) with the composite as the working electrode, a coiled Pt wire (23 cm long and 0.5 mm in diameter) as the counter electrode, and a Ag/AgCl electrode as the reference electrode. In the galvanostatic experiments, the electrochemical cell was fitted with a Teflon cap and a glass-tube with a frit (Bioanalytical Systems, UK) to obtain separate anode and cathode compartments. The latter was done to minimize the changes in the pH within the working electrode compartment as a result of the oxidation and reduction of water taking place on the Pt counter electrode. A schematic diagram of the electrochemical cell used can be found in the *Figure S1*.

The composite paper sheets (used as the working electrodes) were cut into rectangular pieces with the approximate dimensions 1.1×0.5×0.1 cm^3^, corresponding to a weight of about 20 mg. The composites were contacted and immersed in the electrolyte using a Pt-wire, which was coiled around the composite to obtain a good ohmic contact. Fresh composites were used for each measurement, and separate cells were used for the batch-wise extraction and release experiments, respectively.

##### 2.2.1.1 Pre-treatment of PPy-cellulose composite

Prior to the galvanostatic extractions, all composites were first reduced at −0.5 V for 100 s in 10 mL of 2.0 M NaCl.

##### 2.2.1.2. Time dependent extraction of DNA

a) Extraction of DNA oligomers in the absence of applied current: Prior to the experiments, each composite was first soaked for 10 min in 10 mL of the PBS buffer. The composite was then moved into 10 mL of the extraction solution containing (dT)_6_ oligomers tagged with 6-FAM fluorophore and was kept there for varying time intervals, i.e. 600 to 4000 s. The stock solution of 100 µM of (dT)_6_ oligomers tagged with 6-FAM used in these experiments was prepared according to the manufacturer's instructions (Biomers.net, Germany). The extraction solution was prepared by diluting 100 µL of stock solution with 10 mL of PBS. The final concentration of the oligomers in the solution was thus 1±0.002 µM.

b) Constant-current controlled extraction of DNA: Following the pre-treatment (i.e. 100 s reduction at −0.5 V in 10 mL of 2.0 M NaCl), the composite was immersed in 10 mL of the extraction solution containing (dT)_6_ oligomers tagged with 6-FAM fluorophore. The composite was then oxidized by applying a constant anodic current of 1.2 mA. The extraction (i.e. oxidation) time was varied between 600 and 4000 s. The extraction solution was prepared as described above for the extractions in the absence of an applied current.

##### 2.2.1.3. Cumulative constant-time galvanostatic extraction and release of DNA oligomers

In these experiments, the composites were placed in 10 mL of the extraction solution containing (dT)_6_ oligomers tagged with 6-FAM (after the reductive pre-treatment) and oxidized by applying a constant current of 1.2 mA for 800 s. Prior to the release (i.e. the reduction of the PPy), the oxidized composite was rinsed with fresh 10 mL PBS solution and subsequently with deionized water to remove non-specifically bound DNA oligomers, i.e. DNA oligomers retained due to the extensive internal porosity of the composite (the so-called sponge effect). The composite was then immersed in 10 mL borax buffer solution, which originally did not contain any DNA, and the composite was reduced by applying a constant current of −1.6 mA for 2000 s at a temperature of 70 °C. The larger reduction charge (1.6 mA×2000 s = 3200 mC) compared to the oxidation charge (1.2 mA×800 s = 960 mC) was employed to ensure that the reduction of the composite was as complete as possible since it is well-known [45] that the reduction of a PPy film is significantly slower than the corresponding oxidation.

Four consecutive measurements with fresh composites were performed for both the extraction and release in the respective solutions. As described above, all composites were thoroughly rinsed with PBS buffer and subsequently with deionized water when switching between the extraction and release cells.

##### 2.2.1.4 Cumulative constant-time galvanostatic extraction and release of DNA mixtures

The extraction solution was in this case prepared by mixing 50 µL of 100 µM (dT)_6_ 6-FAM, (dT)_20_ Texas Red and (dT)_40_ Cy3 oligomers, respectively, in 10 mL of PBS. The molecular ratio of the oligomers (dT)_6_: (dT)_20_: (dT)_40_ in the extraction solution was thus 1∶1∶1. The release experiments were performed in 10 mL of borax buffer which originally did not contain any DNA oligomers. The reduced composites were immersed in the extraction solution as described above and oxidized by applying a constant current of 1.2 mA for 800 s. The composite was then transferred to the release cell and was reduced by applying a constant current of −1.6 mA for 2000 s at a temperature of 70 °C.

Four consecutive measurements with fresh composites were performed for both the extraction and release in the respective solutions. The composites were rinsed with fresh 10 mL PBS between the extraction and release procedures.

#### 2.2.2. Fluorescence measurements

The changes in the DNA concentrations of the solutions following the electrochemical extractions and release experiments were monitored using a spectrofluorometer (TECAN Infinite M200, Austria). Black Corning 96-well flat plates (Corning, Lowell, MA) were used, and the gain-value was fixed at 100 in all measurements. Three 100 µL solution samples were taken for each analysis. For the determination of the galvanostatic extraction and release yield in the (dT)_6_ tagged 6-FAM oligomer experiments, as well as of the extraction experiments involving the mixtures of the 6-FAM, Texas Red and Cy3 tagged oligomers, an excitation wavelength of 460 nm was used while the emission spectrum was recorded between 505 and 560 nm. For the release experiments involving the 6-FAM, Texas Red and Cy3 fluorophore-tagged DNA, different emission and excitation wavelengths were used depending on the fluorophore. The excitation wavelengths 460, 573, and 523 nm and emission spectral ranges 505–560, 600–650 and 556–586 nm were thus used for 6-FAM, Texas Red and Cy3 fluorophores, respectively. Calibration curves were constructed for each specific fluorophore based on the respective gain-values, excitation wavelengths, and emission spectra.

## Results and Discussion

The highly porous conductive PPy-cellulose composite material used in this study consists of black paper sheets with a large internal specific surface area (see *Figure S2*), which can be directly immersed in the DNA oligomer buffer solution for batch-wise extractions. Numerous studies in the past have shown that DNA molecules, which are negatively charged polyelectrolytes, adsorb on the surface of (partially oxidized) PPy particles [30]–[33], [35], [37]–[39]. In particular, it has been shown that DNA oligomers can penetrate inside PPy films thanks to the presence of an extensive channel network [39]. However, as the adsorption times used in these studies generally were of the order of hours, it is, interesting to study if electrochemically controlled extraction of DNA can provide similar extraction yields in significantly shorter times using the present highly porous composite material. It is likewise very interesting to study if the previously found [37] low release rate of the extracted DNA can be increased by actively driving the reduction of the PPy with an applied cathodic current and the use of 30–50 nm thin PPy coatings.

**Figure 1:**
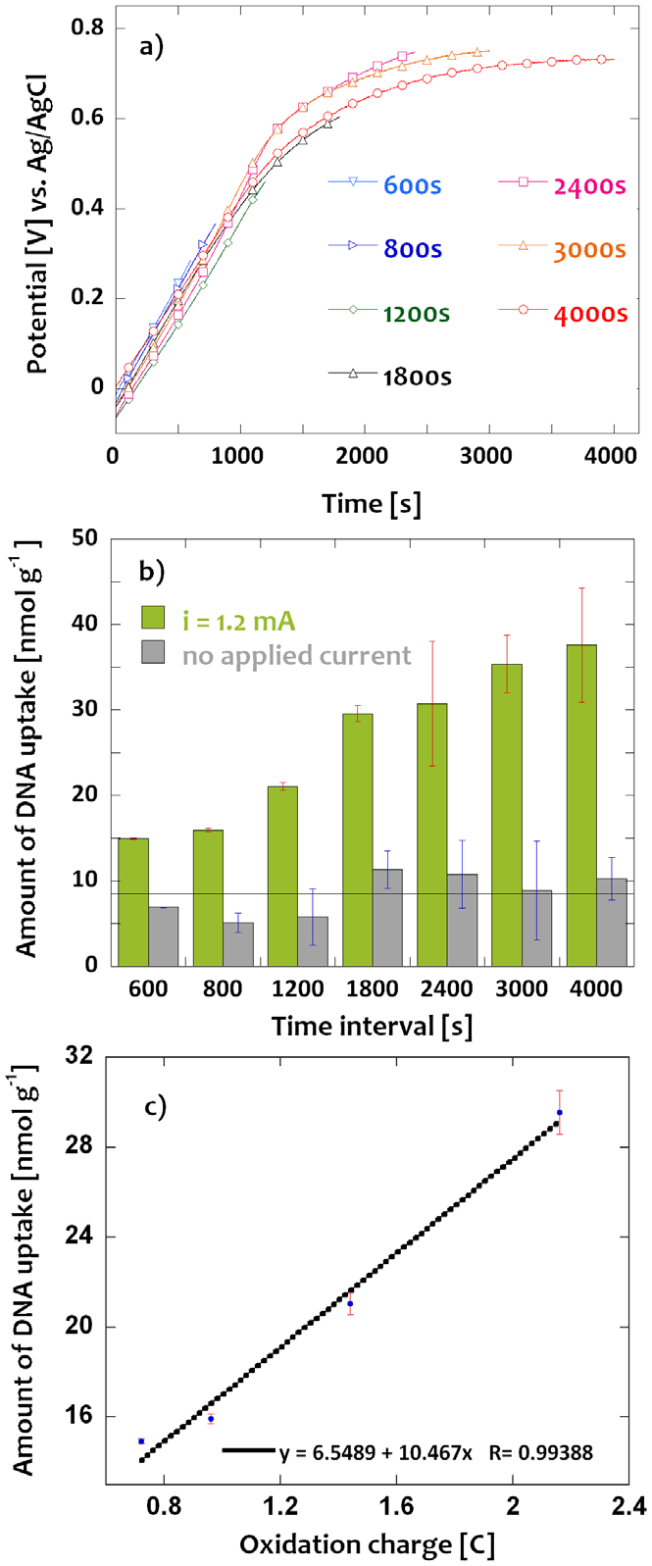
Time dependent extraction of tagged (dT)_6_ oligomers from a 1 µM solution employing a constant anodic current of 1.2 mA: a) Potential of the PPy-cellulose composite versus time and b) tagged (dT)_6_ oligomer uptake during different time intervals with no applied current and an applied current of 1.2 mA, respectively: the dashed line represents the average uptake during all intervals with no applied current c) amount of tagged (dT)_6_ oligomers in the composite as a function of applied charge for the initial four fixed time intervals i.e. (600–1800 s). The results were normalized with respect to the weight of the composite and the error bars represent the standard deviation (n = 3).

Figure 1 shows the results of a galvanostatic experiment, performed to extract the (dT)_6_ oligomers tagged with 6-FAM fluorophore in which a constant current of 1.2 mA was applied to oxidize the PPy-cellulose composite for varying time intervals ranging from 600 to 4000 s. During the oxidation, the PPy chains became positively charged, and anions entered into the film to maintain charge neutrality within the film. As a result of the oxidation, the potential of the PPy composite increased with time as is seen in Figure 1a. In accordance with previous results [47], [49], [50], the potential increased almost linearly with time during the first 1800 s after which a potential plateau of around +0.7 V developed. The latter plateau can be explained by the onset of PPy overoxidation which has been reported [50] to take place at around +0.65 V vs. Ag/AgCl. For longer oxidation times than 1800 s, a significant decrease in the pH of the solution was found which most likely can be explained by the protons liberated during the overoxidation reaction. No such change in the pH was observed for shorter oxidation times. In the subsequent experiments, care was therefore taken to ensure that the potential always was significantly below +0.7 V. In the light of these results, it can thus be assumed that the charge consumed during the 1800 s experiment (viz. 2.16 C) corresponded to the practically available ion extraction capacity of the PPy film. Based on a composite weight of 20 mg, this charge corresponds to an overall ion exchange capacity (for a singly charged anion) of about 1.1 mmol g^−1^. As the latter is about 370 and 30 times larger than the capacities reported by Liljegren et al. [7] and Deinhammer et al. [22], respectively, it is clear that the present material has a much higher capacity than the materials previously employed in conjunction with electrochemically controlled solid phase extraction.

During the oxidation of PPy, any negatively charged species can potentially enter as counter ions within the PPy film. Since the experiments were carried out in the presence of an excess of buffer ions, it is reasonable to assume that mainly the buffer anions were involved in the charge compensation step and that the DNA subsequently replaced some of the buffer ions in an ion exchange process. The apparent DNA exchange capacity will therefore depend on the concentrations and type of all other competing ions present in the sample solution. To determine the extracted DNA amount, the decrease in the fluorescence intensity of the DNA containing extraction solution was measured after each extraction step. Figure 1b shows the amount of extracted DNA (evaluated from the fluorescence intensity decrease) as a function of time in the absence and presence of an imposed oxidation current of 1.2 mA. In accordance with previous results [30], [32], [33], [35], [37]–[39], (dT)_6_ was extracted also in the absence of a galvanostatic oxidation of the PPy composite. The latter can be explained by two effects, viz. (i) the retention of liquid containing (dT)_6_ in the voids between composite nanofibers of the high surface area conductive paper materials (see below) and (ii) the fact that the PPy composite already was partially oxidized as a result of the manufacturing process and therefore should have a certain anion exchange capacity. More importantly, the results in Figure 1b clearly show that the ion exchange capacity of the PPy composite can be increased significantly by oxidizing the PPy using a constant current. With an oxidation time of 1800 s (i.e. an oxidation charge of 2.16 C), the amount of extracted DNA was thus about 3.5 times larger than in the absence of the oxidation current (the horizontal line, at around 8.4 nmol g^−1^, in Figure 1b represents the average uptake level of (dT)_6_ in the absence of an applied constant current). Another problem with the previously employed [30], [32], [33], [35], [37]–[39] zero oxidation current extraction approach was that the uncertainties in the extracted amounts of DNA were relatively high. This can, at least partially, be ascribed to variations in the oxidation state of the different PPy composites used in the extractions.

For galvanostatic electrochemical extraction of (dT)_6_ at varying time intervals, it is seen in Figure 1b that the amount of extracted DNA oligomers increased during the initial 1800 s, whereas a leveling off effect was observed for longer time intervals. The uncertainty in the number of extracted DNA oligomers for the 2400, 3000, and 4000 s time intervals was rather high due to the interference from the previously mentioned overoxidation of the PPy film and the associated pH changes occurring after about 1800 s. These results support our previous conclusion that extraction times longer than 1800 s (viz. charges higher than 2.16 C) are of little analytical value. As is evident from Figure 1c, the extracted amount of DNA oligomers increased practically linearly with the applied oxidation charge for extraction times up to 1800 s. This indicates that the amount of the extracted DNA oligomers can be controlled by tuning the extraction time, but more importantly that the amount of DNA extracted is proportional to the oxidation time and hence to the positive charge of the PPy composite.

**Figure 2:**
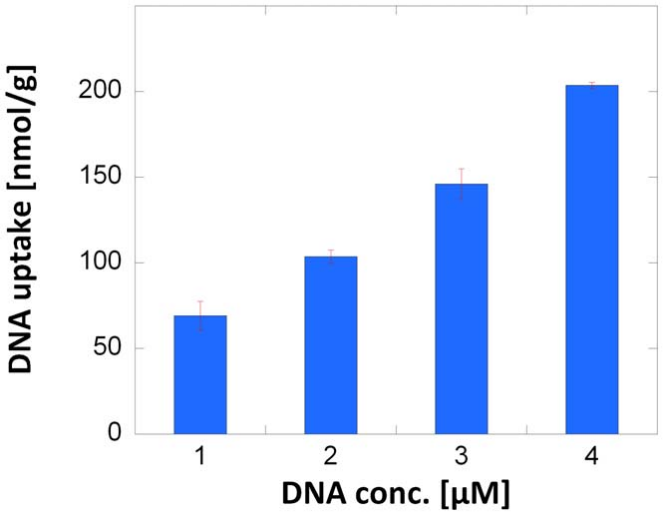
The influence of the DNA oligomer concentration on the extracted amount for galvanostatically controlled extraction (1.2 mA for 800 s) using a PPy-cellulose composite and tagged (dT)_6_ oligomer solutions. The results were normalized with respect to the weight of the composites and the error bars represent the standard deviation (n = 2).

Based on solid phase microextraction theory [20], it is expected that the amount of extracted (dT)_6_ should be proportional to its concentration in the solution. Such a linear relationship was indeed found based on experiments with four different concentrations of (dT)_6_ as is seen in Figure 2. In addition, no significant difference in the extracted amount was found when carrying out the experiments with a 1 µM solution in a buffer with a concentration two times lower than that normally used. The latter is in good agreement with previous results for zero oxidation current adsorption of DNA on PPy [30], [32], [33], [35], [37]–[39]. The fact that no significant difference in the extracted amount was seen in the absence and presence of a 800 s long zero-current period after having oxidized the PPy composite for 800 s using a current of 1.2 mA also indicates that the ion exchange reaction between the DNA and the buffer anions (assumed to be present within the PPy polymer as charge compensating anions) was relatively fast. It should be pointed out that, adsorption times of several hours were generally employed in previous DNA adsorption studies [30], [32], [33], [35], [37]–[39]. We attribute the relative rapid adsorption behavior to the porous structure of the composite and the thin layers of PPy on the high-surface area cellulose matrix. We have previously presented energy filtered TEM data [51] indicating that DNA can access the entire volume of the PPy coatings during the extraction. The present results thus indicate that the amounts of (dT)_6_ within the PPy film after the extraction were determined merely by the concentration of (dT)_6_ in the solution and the oxidation state of the PPy composite.

While Figure 1 and 2 clearly demonstrate the advantages of using the present high-surface area PPy-cellulose composites for direct batch-wise electrochemically controlled solid phase extractions of DNA in the presence of an excess of buffer ions, the success of this approach in biotechnological applications clearly also depends on the possibility of subsequently releasing the extracted DNA by reducing the PPy composite. The latter is (for obvious reasons) not readily studied using the zero current approach since the PPy coating always will remain partially oxidized (i.e. positively charged) in solutions containing oxygen. It has, nevertheless, been found [37] that DNA (adsorbed on partially oxidized PPy coatings) could be released into solutions containing a competing anion at a rate approximately two orders of magnitude lower than that for the adsorption process. Since a reduction of the PPy film removes the positive charge from the polymer, it can be expected that a reduction step should facilitate the DNA release. This is, unfortunately, not necessarily true as it is well-known that sufficiently thick PPy films doped with large anions generally serve as cation exchangers [21], [23], [24], [45], [46], (i.e. that the charge compensation involves ingress of cations rather than release of anions during the reduction of the PPy film). One approach to minimize the latter problem thus involves the use of a very thin PPy coating such as that of the present PPy composite.

**Figure 3:**
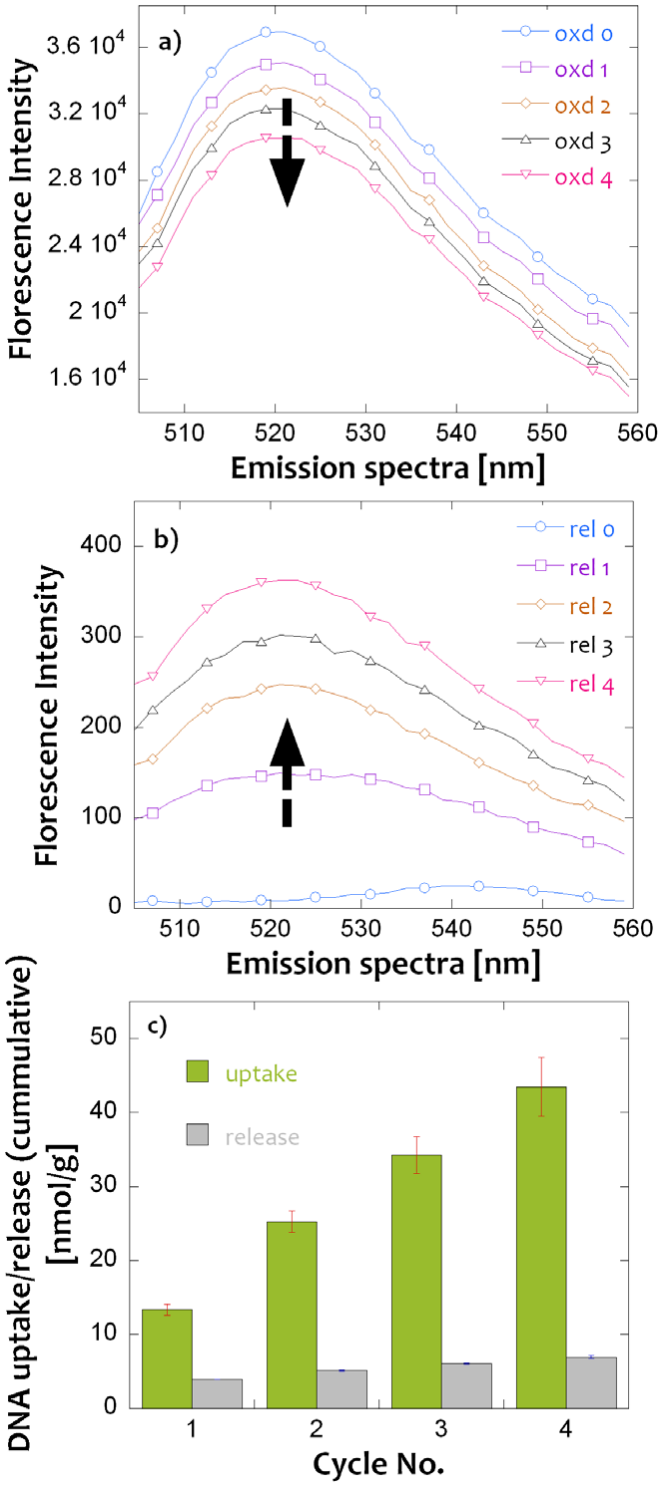
Extraction and release of tagged (dT)_6_ oligomers from a 1 µM solution into pure buffer solutions respectively. Top images: fluorescence emission spectra of tagged (dT)_6_ oligomers following the galvanostatic a) oxidation and b) reduction of the composite for the indicated number of oxidation and reduction cycles. The spectra were recorded in the extraction and the release solutions, respectively. c) Cumulative uptake and release of the (dT)_6_ oligomers following the oxidation and reduction steps. The results have been normalized with respect to weight of the composite and the error bars represent the standard deviation (n = 3).

Figure 3 summarizes the results of batch-wise electrochemical oxidation and reduction experiments carried out with the PPy-cellulose composites in a solution containing (dT)_6_ oligomers. To ensure an as complete reduction as possible, a larger reduction charge, compared to the oxidation charge, was used in these experiments. As can be seen in Figure S3 in the *Supporting Information*, this approach gave rise to hydrogen evolution when the current no longer could be entirely supported by the reduction of the composite. As no attempts to remove oxygen from the release solution were made, oxygen was likewise reduced during the release step. Figure 3a shows that there was a continuous decrease in the fluorescence intensity of the fluorophore tagged (dT)_6_ oligomers in the extraction solution after each oxidation step (the galvanostatic oxidation and reduction curves for four consecutive cycles can be found in the *Figure S3*). Analogously, Figure 3b shows that the fluorescence intensity of the released fluorophore tagged (dT)_6_ oligomers increased steadily from a value close to zero following the electrochemical reductions of the PPy-cellulose composite. The cumulative extracted and released amounts of (dT)_6_ (evaluated from the changes in the fluorescence intensity of the extraction and release solutions) as a function of the number of extraction and release cycles for up to four cycles is shown in Figure 3c. Note that the amount of (dT)_6_ oligomers non-specifically bound to the composite surface and removed in the rinsing step, has been subtracted from the uptake values presented in Figure 3c. This subtracted amount was estimated to be on average 1.20±0.22 nmol g^−1^ (n = 3) under the employed conditions. Note also that the slopes of the calibration curves (see *Figure S4*) for the extraction (from the high oligomer concentration PBS solution) and release (into the low oligomer concentration borax solution) differed significantly, and that the sensitivity was markedly higher in the extraction solution.

Based on the results in Figure 3c, the efficiency of the extraction and release of (dT)_6_ can clearly be derived for the employed experimental conditions. In Figure 3c, it is seen that the extracted amount after one extraction was ∼14 nmol/g while the cumulative amount of extracted oligomers after four consecutive oxidation cycles was ∼44 nmol/g. The latter value, which corresponds to ∼9% of the total amount of (dT)_6_ present in the 10 mL extraction solution, is significantly higher than the previously reported values obtained with electrochemically controlled solid phase extraction [6]–[8], [11], [52], [53]. It should, however, be pointed out that the extraction yield mainly depends on the volume of the (dT)_6_ solution employed in the extraction and that it would be possible to increase the present extraction efficiency significantly by using a different experimental set-up, as was shown by Liljegren and Nyholm [54]. As can be expected since a new composite was used for each extraction, the cumulative extracted amount increased linearly with the number of extractions.

As is also seen in Figure 3c, the electrochemically released amount of (dT)_6_ was about 4 nmol/g after the first reduction cycle and approximately 7 nmol/g (cumulative) after the fourth reduction cycle. These values correspond to an approximate release efficiency of 30% for the first cycle and 16% after four cumulative cycles. The 30% release efficiency for the first cycle is very encouraging as it indicates that a significant release of extracted (dT)_6_ indeed is possible even for release times as short as 2000 s. In the absence of a reduction of the PPy film, it was previously reported [37] that the release rate was approximately two orders of magnitude lower than the extraction rate. The more efficient release in the present case can most likely be ascribed to the reduction of the PPy and the thin PPy layers, both of which should facilitate the release of the extracted DNA. The fact that the release efficiency decreased with increasing number of release cycles indicates that the release of the (dT)_6_ oligomer was controlled by the diffusion of the DNA oligomer into the release solution. The efficiency of such a process will clearly decrease when the concentration in the release solution is increasing. This suggests that the release efficiency ultimately depends on the distribution coefficient for the oligomer with respect to the PPy film and the release solution.

During the reduction of the PPy film, it is reasonable to assume that the charge compensation takes place mainly via insertion of cations and that the released DNA oligomers subsequently diffuse out from the (poorly conducting) reduced PPy film. It is well-known [55] that the PPy film undergoes contraction during the reduction step (and expansion during the oxidation step) which most likely also affects the release of the DNA oligomers. Although release experiments were also performed using a constant potential (rather than a constant current) or with other applied constant currents, the best release yield was observed when employing a constant current of −1.6 mA for 2000 s at 70 °C (i.e. with the conditions used in Figure 3). The higher efficiency at the latter temperature compared to at room temperature supports the hypothesis that the release rate of the DNA oligomers was limited by diffusion. It is possible that the release efficiency could be further increased if a significantly larger cation than Na^+^ was employed in the release solution (it has previously been found that the pH and type of cations in the release solution affect the release of anions from PPy films [52]). This concept was, however, difficult to implement in the present work due to the need for a release solution containing a salt with a sufficiently high solubility, buffer capacity, and conductivity. It should, however, be stressed that the need for a constant pH in the release experiments stemmed merely from the fact that the fluorescence intensities of the chosen fluorophores were pH dependent (see *Figure S5*).

Since the conductivity of a PPy film decreases significantly upon the reduction of the film [49] it may be anticipated that the DNA release efficiency could be increased by increasing the conductivity of the reduced PPy films as this would facilitate a more complete reduction of the PPy film. To test this hypothesis, some experiments were carried out with PPy composites to which chopped carbon fibers (with a diameter of 8 µm) had been included as an additive during the molding of composite paper sheets. With such carbon fiber containing composites, a release yield of 40% was obtained, indicating that the conductivity of the reduced PPy composite indeed is one of the factors controlling the release efficiency.

**Figure 4:**
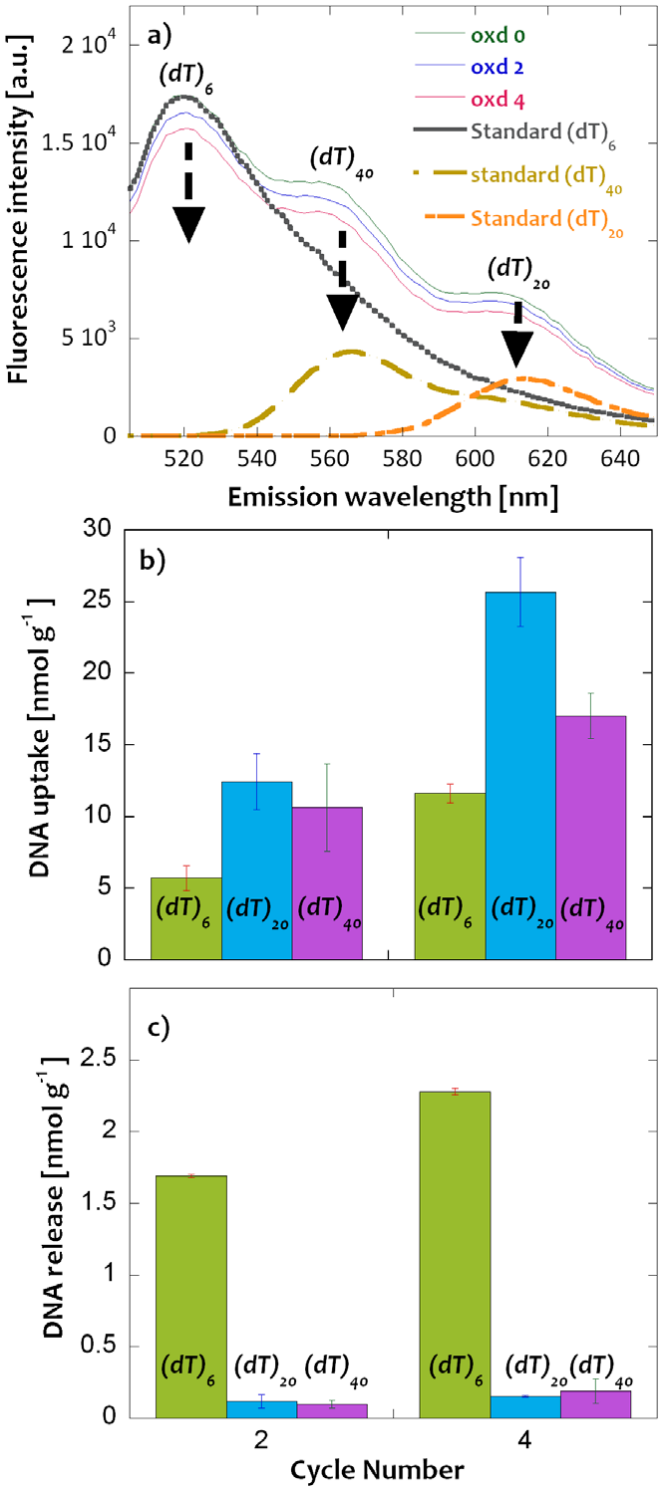
Extraction and release of a mixture of DNA oligomers: a) fluorescence emission spectra of mixtures of tagged (dT)_6_, (dT)_20_ and (dT)_40_ oligomers following galvanostatic extraction for the indicated number of extraction cycles. The emission curves recorded for a concentration of 500 nM of each tagged DNA oligomer are also displayed. Cumulative b) uptake and c) release of the tagged DNA oligomers. The results have been normalized with respect to weight of the composite and the error bars represent the standard deviations (n = 2).

Even though a complete release of the extracted DNA is yet to be demonstrated, the results in Figure 3 clearly show that it is possible to release a significant fraction of the extracted DNA in a relatively short time using galvanostatic reduction of the PPy film. This finding is certainly very encouraging, but additional experiments with larger DNA oligomers are clearly needed to be able to evaluate the general applicability of the method. Figure 4a consequently displays typical fluorescence emission spectra obtained during galvanostatic extraction of DNA oligomers from a solution containing equal concentrations of (dT)_6_, (dT)_20_ and (dT)_40_ tagged with 6-FAM, Texas Red and Cy3 fluorophore, respectively, after four consecutive extraction cycles. In analogy with the (dT)_6_ results in Figure 3, it is seen that the fluorescence intensities decreased for all three DNA oligomers after each extraction cycle. Figure 4b shows the cumulative number of DNA oligomers extracted from the solution after two and four cycles, respectively. Although it is clearly seen that all three oligomers were extracted during the oxidation of the PPy film, it is difficult to draw any definitive conclusions regarding any preferential extraction of any of the oligomers due to the uncertainties in the data. The apparent preference for an extraction of the (dT)_20_ oligomer with respect to, at least, the (dT)_6_ oligomer is thus not statistically significant. Significantly different results were, on the other hand, obtained for the oligomers during the subsequent release experiments. In Figure 4c it is clearly seen that only the (dT)_6_ fraction could be released to any substantial extent (the released amounts of the (dT)_20_ and (dT)_40_ oligomers were thus found to be negligible even after four consecutive release cycles). The fact that the (dT)_20_ and (dT)_40_ oligomers could be extracted at least as well as the (dT)_6_ oligomers can be explained by the ion exchange process in which the oligomers replace some of the buffer anions as counter ions in the oxidized PPy film. The finding that only the (dT)_6_ oligomer could be released during the subsequent reduction of the oxidized PPy film suggests that the specific interactions between the oligomers and the PPy composite [37] increase with increasing oligomer size. To be able to release the larger oligomers (and to increase the release efficiency in general) it is thus necessary to overcome these interactions. The latter could possibly be accomplished based on a release approach in which the negatively charged oligomers are driven out of the PPy composite using an external electric field. Such experiments are currently being conducted in our laboratory and the results of these studies will consequently be discussed in a separate communication. The present results hence indicate that it is possible to employ the present PPy-cellulose composite to separate small DNA oligomers from larger ones.

### Conclusions

It has been demonstrated that porous conducting PPy-nanocellulose composites can be used as high-capacity electrochemically controlled solid phase materials for rapid batch-wise extraction and release of (dT)_6_, (dT)_20_, and (dT)_40_ DNA oligomers, respectively. This study constitutes an attempt to design efficient and reversible electrochemically controlled ion exchange membranes suitable for inexpensive batch-wise solid phase extractions of biomacromolecules. It was shown that all three oligomers could be straightforwardly extracted from a solution containing an excess of buffer ions and that a release efficiency of 40% could be obtained for the smallest oligomer. The release efficiency, which to some extent depends on the conductivity of the PPy composite, is most likely controlled by the distribution coefficient for the oligomers with respect to the PPy composite and the release solution. The combination of the use of electrochemically controlled extraction and release experiments and the present porous PPy composite enabled higher and more well-defined ion exchange capacities and faster extractions to be obtained when compared to previous PPy based solid phase extraction approaches. These results indicate that the present combination of high surface area and thin PPy films, yielding both high capacity and rapid access to the PPy layers, could be very promising for the development of inexpensive and efficient electrochemically controlled ion-exchange membranes for batch-wise extraction of a range of different biomolecules.

## Supporting Information

Figure S1 **Schematic diagram of the electrochemical cell used for galvanostatic extraction of DNA oligomers.**
(DOC)

Figure S2 **SEM micrograph of a PPy-cellulose composite also featuring a summary of the primary solid-state characteristics of the sample.** The scale-bar corresponds to 300 nm.
(DOC)

Figure S3 **Four consecutive galvanostatic oxidation (open symbols) and reduction (filled symbols) steps performed with PPy-cellulose composite samples.** The oxidation (extraction step), involved a current of 1.2 mA applied for 800 s in 10 mL PBS buffer containing 1 µ M of (dT)_6_ oligomers tagged with 6-FAM fluorophore. The reduction (release) step consisted of a −1.6 mA current applied for 2000 s in 10 mL borax buffer solution with zero initial concentration of DNA.
(DOC)

Figure S4 **Calibration curves used for the extraction (PBS, pH = 6.8, upper figure) and release (borax, pH = 8.0, lower figure) experiments.** The excitation wavelength of 460 nm was used at gain of 100 and emission spectrum was measured between 505 and 560 nm for different concentrations of (dT)_6_ tagged 6-FAM oligomers.
(DOC)

Figure S5 **Fluorescence intensity versus pH for a PBS solution containing 1** µ**M of the (dT)_6_ tagged 6-FAM oligomers.**
(DOC)
