# Supplementary material for: High-Capacity Conductive Nanocellulose Paper Sheets for Electrochemically Controlled Extraction of DNA Oligomers
Source: PLoS One. 2011 Dec 15;6(12):e29243. doi: 10.1371/journal.pone.0029243 (PMC3240650; doi:10.1371/journal.pone.0029243)
Supplement: Figure S1 — Schematic diagram of the electrochemical cell used for galvanostatic extraction of DNA oligomers. (DOC) [file pone.0029243.s001.doc]

**FIGURE S1**

**High Capacity Conductive Nanocellulose Paper Sheets for Electrochemically Controlled Extraction of DNA Oligomers**

Aamir Razaq^1^, Gustav Nyström^1^, Maria Strømme ^1*^, Albert Mihranyan^1*^, Leif Nyholm^2*^

Figure S1 demonstrates the schematic diagram of the electrochemical cell used. To avoid pH shifts in the bulk solution during electrochemical separations, the counter electrode (CE) was placed inside an electrolyte filled glass tube fitted with a frit. The reference electrode (RE) and the working electrode (WE) were placed directly in the cell beaker filled with the electrolyte.

**
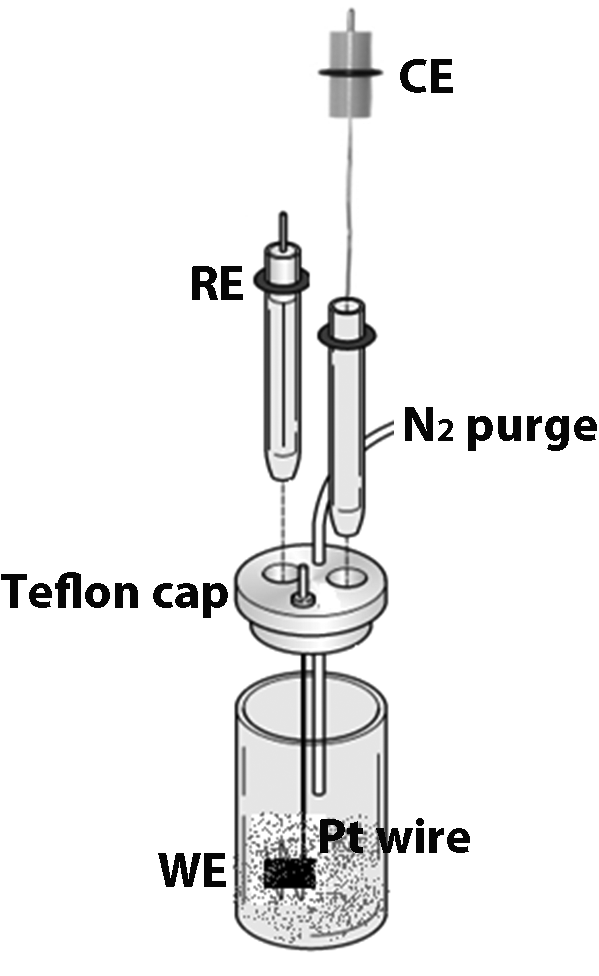
**

*Figure S1. Schematic diagram of the electrochemical cell used for galvanostatic extraction of DNA oligomers.*
