# Supplementary material for: High-Capacity Conductive Nanocellulose Paper Sheets for Electrochemically Controlled Extraction of DNA Oligomers
Source: PLoS One. 2011 Dec 15;6(12):e29243. doi: 10.1371/journal.pone.0029243 (PMC3240650; doi:10.1371/journal.pone.0029243)
Supplement: Figure S2 — SEM micrograph of a PPy-cellulose composite also featuring a summary of the primary solid-state characteristics of the sample. The scale-bar corresponds to 300 nm. (DOC) [file pone.0029243.s002.doc]

**FIGURE S2**

**High Capacity Conductive Nanocellulose Paper Sheets for Electrochemically Controlled Extraction of DNA Oligomers**

Aamir Razaq^1^, Gustav Nyström^1^, Maria Strømme ^1*^, Albert Mihranyan^1*^, Leif Nyholm^2*^

Figure S2 shows the SEM (Leo Gemini 1550 FEG, UK) micrograph together with the summary of the primary solid-state characteristics of the nanocellulose-composite material used. It is seen from the micrograph that the composite material consisted of numerous about 100 nm thick, intertwined nanofibres creating a network of high internal porosity as further supported by the N_2_ gas adsorption BET analysis (ASAP 2020 Micromeritics, USA) and He-pycnometry (AccuPyc 1340 Micromeritics, USA) results. The specific surface area of the composite PPy-cellulose material was found to be 74.6 m^2^ g^-1^. The total porosity of the composite was 78.9 % based the following equation:

$\varepsilon_{\%}=(1-\frac{\rho_{b}}{\rho_{t}})\times100$ (S1)

where ε% is the total porosity, ρ_B_ is the bulk density, and ρ_T_ is the true density. The total pore volume for the pores less than 124 nm was 0.37 cm^3^ g^-1^ as estimated from N_2_ BET gas adsorption isotherms. The electron conductivity of the composite was 1.58 S cm^-1^ as derived from the reciprocal of the slope of the IV-sweep between -1 and +1 V using a semiconductor analyzer device (B1500 A Agilent Technologies, USA) at room temperature.

*Figure S2. SEM micrograph of a PPy-cellulose composite also featuring a summary of the primary solid-state characteristics of the sample. The scale-bar corresponds to 300 nm.*
