# Supplementary material for: High-Capacity Conductive Nanocellulose Paper Sheets for Electrochemically Controlled Extraction of DNA Oligomers
Source: PLoS One. 2011 Dec 15;6(12):e29243. doi: 10.1371/journal.pone.0029243 (PMC3240650; doi:10.1371/journal.pone.0029243)
Supplement: Figure S3 — Four consecutive galvanostatic oxidation (open symbols) and reduction (filled symbols) steps performed with PPy-cellulose composite samples. The oxidation (extraction step), involved a current of 1.2 mA applied for 800 s in 10 mL PBS buffer containing 1 µ M of (dT)6 oligomers tagged with 6-FAM fluorophore. The reduction (release) step consisted of a −1.6 mA current applied for 2000 s in 10 mL borax buffer solution with zero initial concentration of DNA. (DOC) [file pone.0029243.s003.doc]

**FIGURE S3**

**High Capacity Conductive Nanocellulose Paper Sheets for Electrochemically Controlled Extraction of DNA Oligomers**

Aamir Razaq^1^, Gustav Nyström^1^, Maria Strømme ^1*^, Albert Mihranyan^1*^, Leif Nyholm^2*^

Figure S3 depicts the galvanostatic measurements obtained during the oxidation and reduction of the composite employing constant currents of 1.2 and -1.6 mA for 800 and 2000 s, respectively. The (dT)_6_ oligomers tagged with 6-FAM fluorophore were first extracted by oxidizing the PPy film in a PBS solution containing 1 μM of the oligomers. The PPy film was then transferred to a borax buffer solution, which originally did not contain any DNA oligomers, in which the film was reduced. The 800 s oxidation time during the extraction steps (performed at room temperature) was selected so as to keep the sample well below its overoxidation limit, whereas the reduction cycles were performed at 70 ^o^C to facilitate the release of the oligomers by diffusion. As it is seen from Figure S3, the change in the potential was nearly linear for all consecutive oxidation cycles, indicating that the samples were indeed not overoxidized (see the discussion in connection with Figure 1). The fact that the slopes of all the oxidation curves also were very similar even though fresh samples were used for each cycle clearly shows the high reproducibility of the system under the employed experimental conditions. The four consecutive reduction cycles also show an approximately linear dependence of the potential on time for times shorter than about 1800 s after which the potential dropped dramatically as a result of the onset of hydrogen evolution once the PPy film had been reduced.


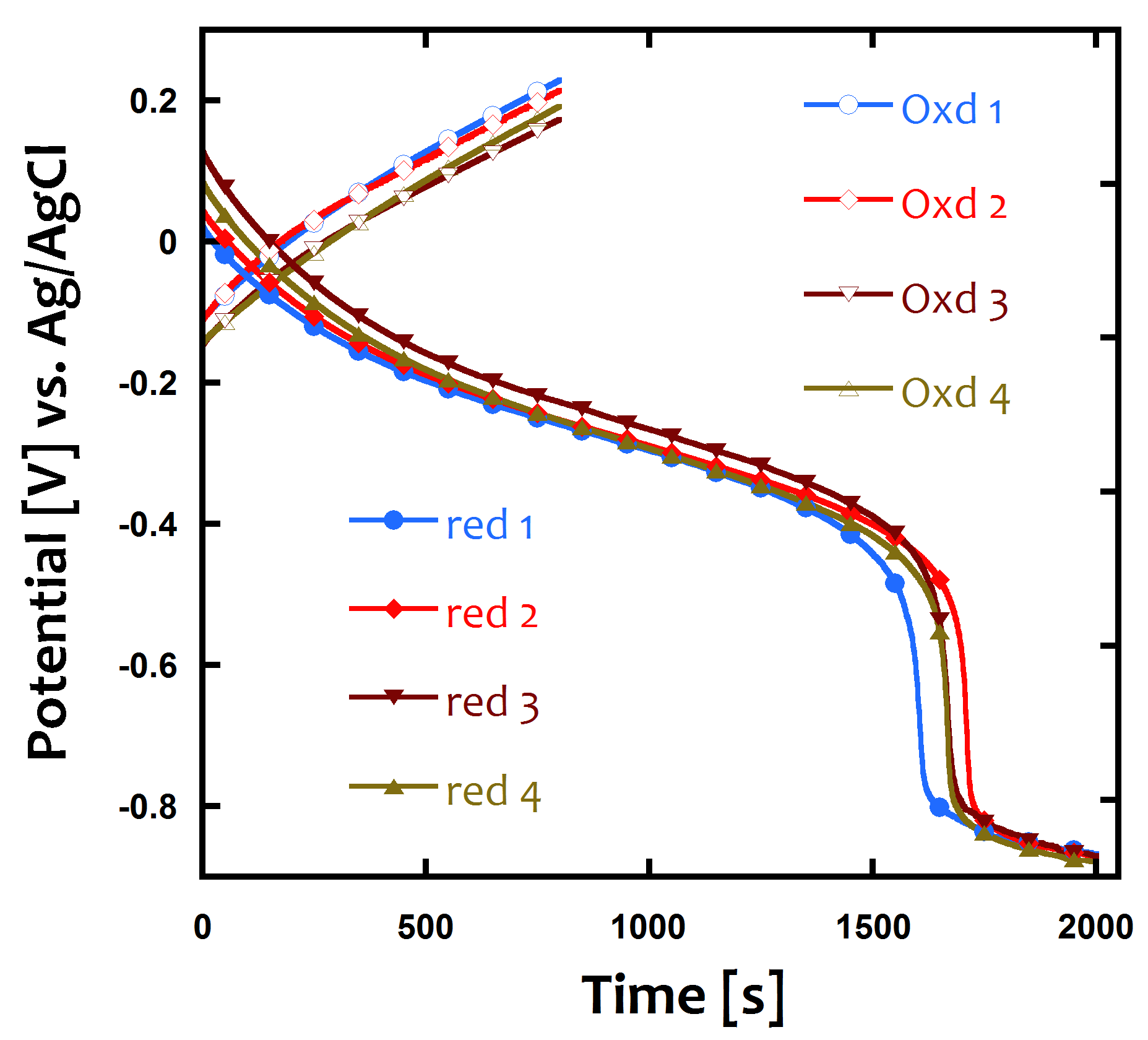


*Figure S3. Four consecutive galvanostatic oxidation (open symbols) and reduction (filled symbols) steps performed with PPy-cellulose composite samples. The oxidation (extraction step), involved a current of 1.2 mA applied for 800 s in 10 mL PBS buffer containing 1 μM of (dT)_6_ oligomers tagged with 6-FAM fluorophore. The reduction (release) step consisted of a -1.6 mA current applied for 2000 s in 10 mL borax buffer solution with zero initial concentration of DNA.*
