# Supplementary material for: High-Capacity Conductive Nanocellulose Paper Sheets for Electrochemically Controlled Extraction of DNA Oligomers
Source: PLoS One. 2011 Dec 15;6(12):e29243. doi: 10.1371/journal.pone.0029243 (PMC3240650; doi:10.1371/journal.pone.0029243)
Supplement: Figure S4 — Calibration curves used for the extraction (PBS, pH = 6.8, upper figure) and release (borax, pH = 8.0, lower figure) experiments. The excitation wavelength of 460 nm was used at gain of 100 and emission spectrum was measured between 505 and 560 nm for different concentrations of (dT)6 tagged 6-FAM oligomers. (DOC) [file pone.0029243.s004.doc]

**FIGURE S4**

**High Capacity Conductive Nanocellulose Paper Sheets for Electrochemically Controlled Extraction of DNA Oligomers**

Aamir Razaq^1^, Gustav Nyström^1^, Maria Strømme ^1*^, Albert Mihranyan^1*^, Leif Nyholm^2*^

Figure S4 represents the calibration curves employed for the calculations of uptake/release of the (dT)_6_ tagged 6-FAM oligomer as showed in Fig. 2c. The calibration curves for the extracted/released solutions were measured at pH of 6.8 and 8.0 respectively. The uptake values presented in Fig. 2c were obtained after the subtraction of amount of the non-specifically (rinsed during washing) adsorbed (dT)_6_ tagged 6-FAM oligomer from the total extracted amount.

*Figure S4. Calibration curves used for the extraction (PBS, pH=6.8, upper figure) and release (borax, pH=8.0, lower figure) experiments. The excitation wavelength of 460 nm was used at a gain of 100 and emission spectra were measured between 505 and 560 nm for different concentrations of (dT)_6_ tagged 6-FAM oligomers.*
