# Supplementary material for: High-Capacity Conductive Nanocellulose Paper Sheets for Electrochemically Controlled Extraction of DNA Oligomers
Source: PLoS One. 2011 Dec 15;6(12):e29243. doi: 10.1371/journal.pone.0029243 (PMC3240650; doi:10.1371/journal.pone.0029243)
Supplement: Figure S5 — Fluorescence intensity versus pH for a PBS solution containing 1 µM of the (dT)6 tagged 6-FAM oligomers. (DOC) [file pone.0029243.s005.doc]

**FIGURE S5**

**High Capacity Conductive Nanocellulose Paper Sheets for Electrochemically Controlled Extraction of DNA Oligomers**

Aamir Razaq^1^, Gustav Nyström^1^, Maria Strømme ^1*^, Albert Mihranyan^1*^, Leif Nyholm^2*^

Figure S5 displays the variation in the fluorescence intensity of (dT)_6_ tagged 6-FAM oligomers caused by a change in the pH of the buffer solution. The 100 μL of 100 μM (dT)_6_ tagged 6-FAM oligomers were diluted in 10 mL buffer solution and pH was altered by drop-wise adding 0.5 M HCl or NaOH, respectively. Separate solutions were prepared to increase and decrease the pH of the buffer solution. The excitation wavelength of 460 nm was use at a gain of 90 and emission spectra were measured between 505 and 600 nm.

***Excitation wavelength: 460 nm***

***Emission range 505-600 nm***

***Gain: 90***

*Figure S5 Fluorescence intensity versus pH for a PBS solution containing 1 μM of the (dT)_6_ tagged 6-FAM oligomers.*
